# A Tumor Suppressor Gene-Based Prognostic Classifier Predicts Prognosis, Tumor Immune Infiltration, and Small Molecule Compounds in Breast Cancer

**DOI:** 10.3389/fgene.2021.783026

**Published:** 2022-02-03

**Authors:** Suxiao Jiang, Xiangjing Bu, Desheng Tang, Changsheng Yan, Yan Huang, Kun Fang

**Affiliations:** ^1^ Department of Surgery, Yinchuan Maternal and Child Health Hospital, Yinchuan, China; ^2^ Department of Surgery, The First Affiliated Hospital of Harbin Medical University, Heilongjiang, China; ^3^ Department of Surgery, Affiliated Hospital of Ningxia Medical University, Ningxia, China

**Keywords:** breast cancer, tumor suppressor genes, prognostic classifier, clinical outcomes, immune infiltration, small molecule agents

## Abstract

**Objective:** Tumor suppressor genes (TSGs) play critical roles in the cell cycle checkpoints and in modulating genomic stability. Here, we aimed to develop a TSG-based prognostic classifier for breast cancer.

**Methods:** Gene expression profiles and clinical information of breast cancer were curated from TCGA (discovery set) and Gene Expression Omnibus (GEO) repository (GSE12093 and GSE17705 datasets as testing sets). Univariate cox regression analysis and random forest machine learning method were presented for screening characteristic TSGs. After multivariate cox regression analyses, a TSG-based prognostic classifier was constructed. The predictive efficacy was verified by C-index and receiver operating characteristic (ROC) curves. Meanwhile, the predictive independency was assessed through uni- and multivariate cox regression analyses and stratified analyses. Tumor immune infiltration was estimated *via* ESTIMATE and CIBERSORT algorithms. Small molecule agents were predicted through CMap method. Molecular subtypes were clustered based on the top 100 TSGs with the most variance.

**Results:** A prognostic classifier including nine TSGs was established. High-risk patients were predictive of undesirable prognosis. C-index and ROC curves demonstrated its excellent predictive performance in prognosis. Also, this prognostic classifier was independent of conventional clinicopathological parameters. Low-risk patients exhibited increased infiltration levels of immune cells like T cells CD8. Totally, 48 small molecule compounds were predicted to potentially treat breast cancer. Five TSG-based molecular subtypes were finally constructed, with distinct prognosis and clinicopathological features.

**Conclusion:** Collectively, this study provided a TSG-based prognostic classifier with the potential to predict clinical outcomes and immune infiltration in breast cancer and identified potential small molecule agents against breast cancer.

## Introduction

Breast cancer represents the most frequently diagnosed malignancy among women globally, with an estimated annual death of 41,760 cases (DeSantis et al., 2019; Siegel et al., 2020). Despite the much progress in early detection, diagnostic and therapeutic schemes, relapse, distant metastases, and resistance remain common (Jabbarzadeh Kaboli et al., 2020; Lim et al., 2020). This malignancy is not a single disease, but a heterogeneous and diverse population (Xie et al., 2019). Patients at similar histological stages have distinct clinical characteristics, responses to integrated treatments as well as prognosis. The study of the molecular complexity prompts us to comprehensively probe ways for better identifying high-risk patients (Li et al., 2020). Research has shown that polygenic features may become more precise compared with traditional methods in terms of risk stratification (Li et al., 2019). Therefore, in-depth research is urgently required for unraveling the mechanisms underlying as well as studying robust prognostic classifier for breast cancer.

Tumorigenesis is a multi-step process, which can be attributed to the gain-of-function mutations of oncogenes as well as the loss-of-function mutations of tumor suppressor genes (TSGs) (Chen et al., 2020). Intuitively, inhibition of activated oncogenes is easier than restoration of the function of inactivated TSGs (Chen et al., 2020). Despite this, modulating dysregulated TSGs is equally important for carcinogenesis (Gerstung et al., 2020). TSGs play critical roles in the cell cycle checkpoints as well as in maintaining genomic stability (Kontomanolis et al., 2020). A few potential therapeutic schedules for TSGs or pathways controlled by these genes have emerged in breast cancer (Choi et al., 2014; Gianni et al., 2018; O’Leary et al., 2018). Based on the characteristic TSGs, we developed and externally verified a prognostic classifier for breast cancer that was capable of predicting prognosis and immune infiltration as well as screening promising small molecule agents by applying bioinformatics and machine learning methods. Thus, our findings may offer novel clues and landscape concerning the prognostic evaluation of this malignancy.

## Materials and Methods

### Data Acquisition and Preprocessing

In total, 1,217 human TSGs (Supplementary Table S1) were downloaded from the Tumor Suppressor Gene database (TSGene; version 2.0; http://bioinfo.mc.vanderbilt.edu/TSGene/) (Zhao et al., 2016). Breast cancer datasets were searched from the Cancer Genome Atlas (TCGA; https://portal.gdc.cancer.gov/) and Gene Expression Omnibus (GEO) repository (https://www.ncbi.nlm.nih.gov/gds/). The inclusion criteria of samples were as follows: 1) histologically diagnosed with malignant breast cancer; 2) available transcriptome data; and 3) available follow-up information. Totally, RNA-seq profiles, clinicopathological parameters, and prognostic information of 1,059 breast cancer patients were included from TCGA cohort *via* TCGAbiolink package (Colaprico et al., 2016). Meanwhile, RNA-seq data of 291 normal breast tissues were also retrieved from TCGA cohort. Microarray expression profiling and survival information of 136 breast cancer who received adjuvant tamoxifen therapy was curated from the GSE12093 (Zhang et al., 2009). Meanwhile, we gathered gene expression profiles and follow-up data of 298 ER-positive breast cancer patients who uniformly experienced tamoxifen treatment for 5 years from the GSE17705 dataset (Symmans et al., 2010). The GSE12093 and GSE17705 datasets were both based on the platform of GPL570 Affymetrix Human Genome U133A Array. Batch effects were corrected utilizing ComBat function of sva package (Leek et al., 2012). RNA-seq FPKM value was transformed to TPM format. Microarray data were normalized utilizing Robust MultiChip Analysis (RMA) method (Irizarry et al., 2003), followed by quantile standardization. The expression value was then log2 converted. The probes were mapped to gene symbols in line with the GPL570 annotation files. For making the expression level genes comparable, the expression value of each gene was standardized with Z-score conversion. Here, TCGA cohort was used as a discovery set and the GSE12093 and GSE17705 datasets were utilized as testing set.

### Identification of Characteristic Tumor Suppressor Genes

Univariate cox regression models were conducted to evaluate the associations between TSGs and overall survival (OS) across breast cancer patients in discovery set. TSGs with *p* < 0.05 were screened as prognostic genes. These prognostic TSGs were ordered with random survival forest package (Wang and Zhou, 2017). The analysis was run with the number of Monte Carlo iterations of 100 and the number of steps forward of 5 [14]. Here, TSGs with relative importance >0.4 were considered as characteristic variables.

### Development of a Prognostic Classifier Based on Tumor Suppressor Genes

Multivariate cox regression model was conducted for establishing a prognostic classifier based on the characteristic TSGs in the discovery set. The risk scoring system was developed in line with the following formula: risk score (RS) = coefficient of TSG 1 * expression of TSG 1 + coefficient of TSG 2 * expression of TSG 2+…+ coefficient of TSG *n* * expression of TSG *n*. The RS of each patient was determined according to the scoring formula. The patients were stratified into high- and low-risk subgroups with the median RS as the cutoff value. The mRNA expression of the characteristic TSGs was visualized into a heat map *via* pheatmap package. Kaplan–Meier curves and log-rank test were utilized for comparing the OS difference between subgroups *via* survival package. Through survivalROC package (Lorent et al., 2014), the area under the curve (AUC) and the best cutoff were generated by the time-dependent receiver operating characteristic (ROC) for verifying the performance of this prognostic classifier in predicting OS. Furthermore, C-index was calculated for evaluating the probability of the concordance between TSG-based prognostic classifier-predicted and actual survival utilizing survcomp package.

### Independent Prognostic Analysis

Uni- and multivariate Cox regression analyses were conducted whether the TSG-based prognostic classifier was independent of clinicopathological parameters (age, T, N, M, and stage) in the discovery set. Hazard ratio, 95% confidence interval (CI), and *p*-value were determined in each parameter. The AUC values were compared between the TSG-based prognostic classifier and other clinicopathological parameters.

### Stratified Analysis

Stratified analysis was carried out on the basis of clinicopathological parameters covering age, T, N, M, and stage. Kaplan–Meier curves of OS and log-rank test were presented for assessing the predictive efficacy of the TSG-based prognostic classifier in different subgroups.

### External Validation of the Tumor Suppressor Gene-Based Prognostic Classifier

With the same formula, the RSs of breast cancer patients were calculated in the GSE12093 and GSE17705 sets. Patients were separated into high- and low-risk subgroups with the median RS. The predictive performance of the TSG-based prognostic classifier was verified by log-rank test and ROC curves.

### Analyses of the Expression and Prognosis of Characteristic Tumor Suppressor Genes

The mRNA expression of each characteristic TSG in the TSG-based prognostic classifier was compared in 1,085 breast cancer tissues and 291 normal breast tissues in TCGA dataset with Wilcoxon test. Kaplan–Meier curves of OS and log-rank test were utilized for investigating the prognostic implication of the characteristic TSGs across breast cancer patients.

### Gene Set Enrichment Analysis

GSEA computational method (Subramanian et al., 2005) was utilized for comparing the enrichment differences of gene sets between high- and low-risk subgroups based on gene expression profiling. Kyoto Encyclopedia of Genes and Genomes (KEGG) gene sets were curated from the Molecular Signatures database (https://www.gsea-msigdb.org/gsea/msigdb) (Liberzon et al., 2015). Pathways with |normalized enrichment score (NES)| ≥2, nominal *p*-value <0.05, and false discovery rate (FDR) <0.05 were significantly enriched.

### Analysis of the Overall Infiltration of Immune and Stromal Cells

Estimation of STromal and Immune cells in MAlignant Tumors using Expression data (ESTIMATE) algorithm was employed for inferring the overall infiltration levels of immune cells (immune score) and stromal cells (stromal score) in breast cancer specimens in TCGA cohort according to the mRNA expression profiles (Yoshihara et al., 2013).

### Estimation of the Composition of Tumor-Infiltrating Immune Cells

Cell type Identification By Estimating Relative Subsets Of RNA Transcripts (CIBERSORT) deconvolution algorithm (http://cibersort.stanford.edu/) was applied for quantifying the composition of 22 tumor-infiltrating immune cells in breast cancer tissues in TCGA dataset (Newman et al., 2015). This analysis was run on 1,000 permutations based on the normalized gene expression profiling and the samples were screened in line with *p*-value <0.05. The LM22 feature matrix was curated as a reference set. The results were visualized *via* vioplot and corrplot packages.

### Prediction of Small Molecule Agents

To explore TSG-based prognostic classifier-relevant genes, differential expression analyses were carried out between high- and low-risk subgroups in TCGA dataset by limma package with the cutoff of adjusted *p*-value <0.05 (Ritchie et al., 2015). The top 200 upregulated genes and the top 200 downregulated genes were separately analyzed by the connectivity map (CMap; http://portals.broadinstitute.org/cmap/) project (Lamb et al., 2006). Connectivity scores ranging from −1 to 1 were determined for estimating the connection between compounds and the query signature. Negative score was indicative the query signature might be suppressed by a specific agent. Meanwhile, positive score was indicative that the query signature might be promoted by a specific agent. Small molecule agents with *p*-value <0.05 might potentially treat breast cancer. Mode-of-action (MoA) analyses were utilized for exploring shared mechanisms of action among candidate agents.

### Consensus Clustering Analyses

Consensus clustering analyses were presented through the ConsensusClusterPlus package across breast cancer patients in TCGA cohort based on the mRNA expression profiling of the top 100 TSGs with the most variance (Wilkerson and Hayes, 2010). This analysis was run with 50 iterations and resample rate of 80%. Principal component analysis (PCA) was used for verifying this clustering. The OS differences were compared with Kaplan–Meier curves and log-rank test.

### Statistical Analysis

All statistical tests were conducted *via* R software (version 3.6.1; https://www.r-project.org) and appropriate packages. Continuous variables were compared using Student’s t-test or Wilcoxon test. Moreover, categorical variables were compared through Chi-square test. The *p*-value indicated statistical significance.

## Results

### Development of a Tumor Suppressor Gene-Based Prognostic Classifier for Breast Cancer

**FIGURE 1:**
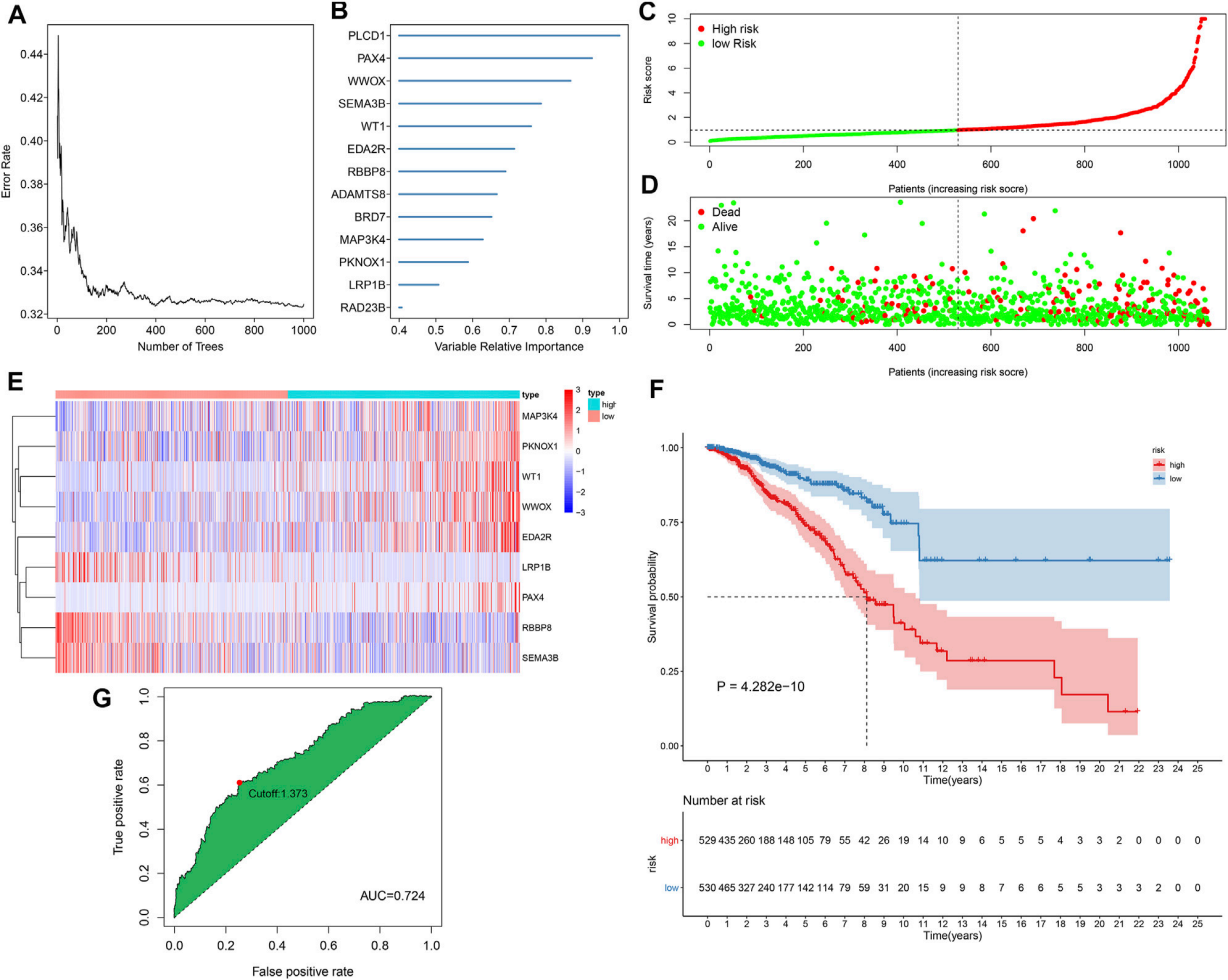
Development of a tumor suppressor gene (TSG)-based prognostic classifier for breast cancer in the Cancer Genome Atlas (TCGA) cohort. **(A)** Correlation between number of classification trees and error rate. **(B)** The rank of characteristic TSGs according to the relative importance. **(C)** The determination of high- and low-risk subgroups according to the median risk score (RS) (vertical dotted line). **(D)** Distribution of survival status in high- and low-risk subgroups. **(E)** Heat map visualizing the mRNA expression of each characteristic TSG in high- and low-risk subgroups. **(F)** Kaplan–Meier curves of overall survival (OS) and log-rank test of high- and low-risk subgroups. **(G)** Receiver operating characteristic (ROC) curve of the TSG-based prognostic classifier.

This study gathered 1,217 TSGs from the TSGene database. Among them, 116 TSGs displayed significant associations with the OS of breast cancer in TCGA dataset (Supplementary Table S2). By random forest method, 13 characteristic TSGs with relative importance >0.4 were selected in TCGA dataset, including PLCD1, PAX4, WWOX, SEMA3B, WT1, EDA2R, RBBP8, ADAMTS8, BRD7, MAP3K4, PKNOX1, LRP1B, and RAD23B (Figures 1A, B). Nine TSGs with non-zero coefficient were included for constructing the multivariate cox regression model (Table 1). Combining the coefficients and expression of above TSGs, the prognostic classifier was developed in the discovery set and the RS of each patient was calculated. According to the median RS, breast cancer patients in the discovery set were stratified into high- and low-risk subgroups (Figure 1C). Furthermore, we observed that there were more patients with dead status in high-risk subgroup compared with low-risk subgroup (Figure 1D). In Table 2, age and T were significantly correlated to the TSG-based prognostic classifier in the discovery set. Heat map showed the differences in mRNA expression of each characteristic TSG between high- and low-risk subgroups (Figure 1E). In Figure 1F, we found that low-risk patients had the distinct survival advantage compared with high-risk patients. The C-index (0.708) and ROC curves (AUC = 0.724 and cutoff = 1.373) were indicative of the well predictive performance of this TSG-based prognostic classifier (Figure 1G).

**TABLE 1:** Coefficients of nine tumor suppressor genes (TSGs) in the multivariate Cox regression model.

TSGs	Coefficient	HR	HR.95L	HR.95H	*p*-Value
EDA2R	0.668266	1.950851	1.544136	2.464691	2.12E−08
LRP1B	−0.24773	0.780573	0.588038	1.036148	0.086484
MAP3K4	0.347939	1.416145	0.880307	2.278145	0.151458
PAX4	5.92132	372.9037	32.50129	4,278.513	1.97E−06
PKNOX1	0.504298	1.655823	0.920447	2.978716	0.092323
RBBP8	−0.44954	0.637925	0.516784	0.787463	2.87E−05
SEMA3B	−0.19055	0.826503	0.707583	0.96541	0.016212
WT1	0.267554	1.306765	1.141355	1.496147	0.000107
WWOX	0.354674	1.425716	1.206859	1.684262	3.03E−05

**TABLE 2:** Clinicopathological characteristics of high- and low-risk subgroups in the discovery set.

Characteristics		High-risk group (*n* = 529)	Low-risk group (*n* = 530)	Total (*n* = 1,059)	*p*-Value
Age	<65	336	405	741	6.43E−06
	≥65	193	125	318	
	Stage I	83	96	179	
	Stage II	293	306	599	
Stage	Stage III	126	113	239	1.64E−01
	Stage IV	14	6	20	
	NA	13	9	22	
	T1	124	153	277	
	T2	317	294	611	
T	T3	59	71	130	0.008319
	T4	27	11	38	
	N/A	2	1	3	
	M0	438	447	885	
M	M1	15	7	22	1.30E−01
	N/A	76	76	152	
	N0	252	244	496	
	N1	160	194	354	
N	N2	67	52	119	1.48E−01
	N3	38	35	73	
	N/A	12	5	17	

### The Tumor Suppressor Gene-Based Prognostic Classifier is Independent of Conventional Clinicopathological Parameters

**FIGURE 2:**
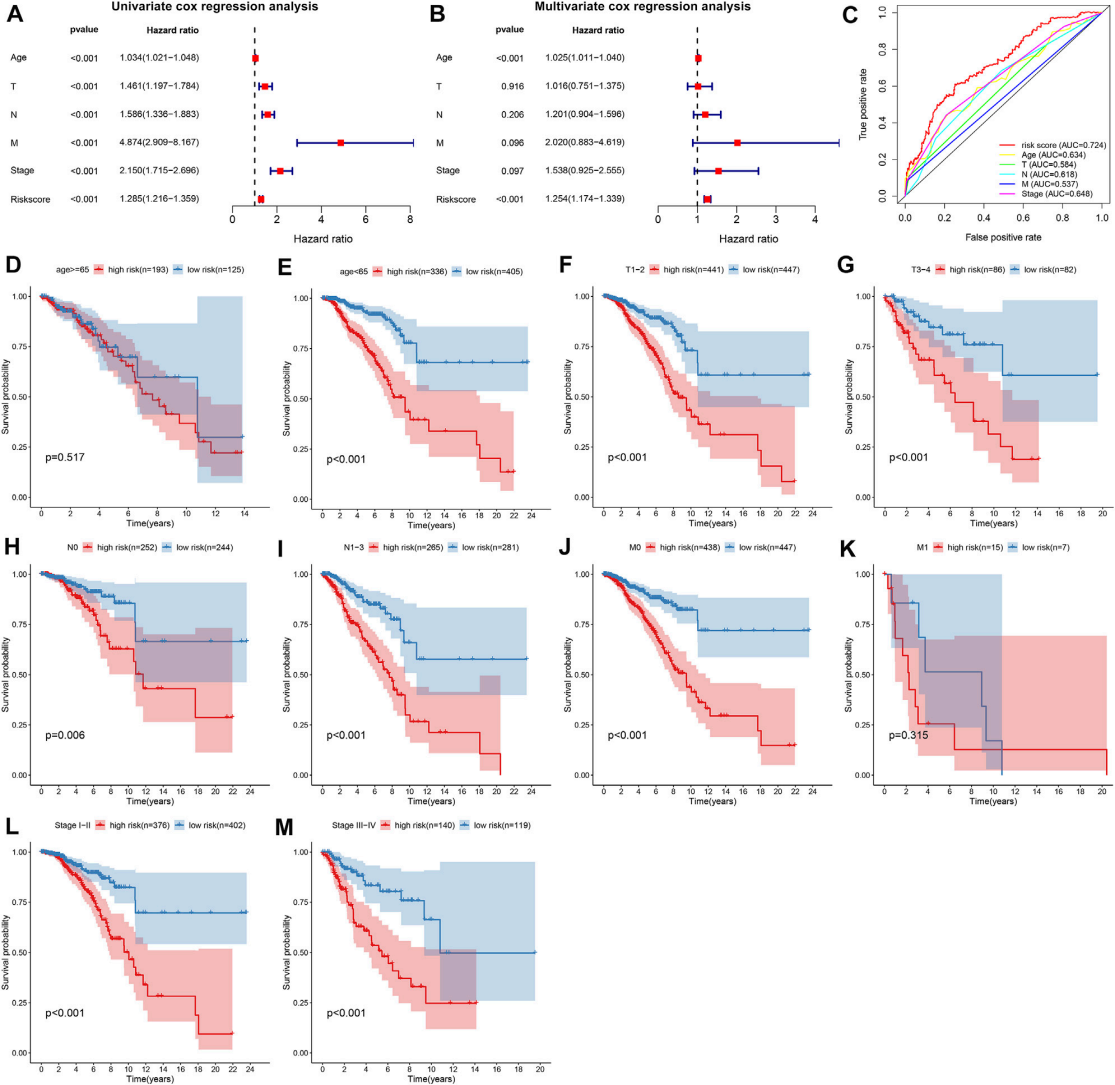
The TSG-based prognostic classifier is independent of conventional clinicopathological parameters across breast cancer in TCGA dataset. **(A,B)** Uni- and multivariate Cox regression models of the associations between age, T, N, M, stage, and RS and OS of breast cancer patients. **(C)** Comparison of the area under the curve (AUCs) of age, T, N, M, stage, and RS. Kaplan–Meier curves and log-rank tests of high- and low-risk breast cancer patients at different subgroups according to clinicopathological parameters, including **(D,E)** age ≥65 and <65; **(F,G)** T1-2 and T3-4; **(H,I)** N0 and N1-3; **(J,K)** M0 and M1; **(L,M)** stages I–II and stages III–IV.

As shown in univariate cox regression models, age, T, N, M, stage, and TSG-based prognostic classifier were all risk factors of breast cancer prognosis in TCGA dataset (Figure 2A). Further multivariate cox regression models uncovered that the TSG-based prognostic classifier was independent of the above clinicopathological parameters (Figure 2B). Compared with conventional clinicopathological parameters, the TSG-based prognostic classifier had the highest AUC value (Figure 2C), indicating this prognostic classifier was superior to these clinicopathological parameters in predicting prognosis. Stratified analysis uncovered that high RS was indicative of poorer OS than low RS at different subgroups according to clinicopathological parameters, including age ≥65 and <65 (Figures 2D, E), T1-2 and T3-4 (Figures 2F, G), N0 and N1-3 (Figures 2H, I), M0 and M1 (Figures 2J, K), and stages I–II and stages III–IV (Figures 2L, M).

### External Verification of the Predictive Performance of the Tumor Suppressor Gene-Based Prognostic Classifier in Breast Cancer Prognosis

**FIGURE 3:**
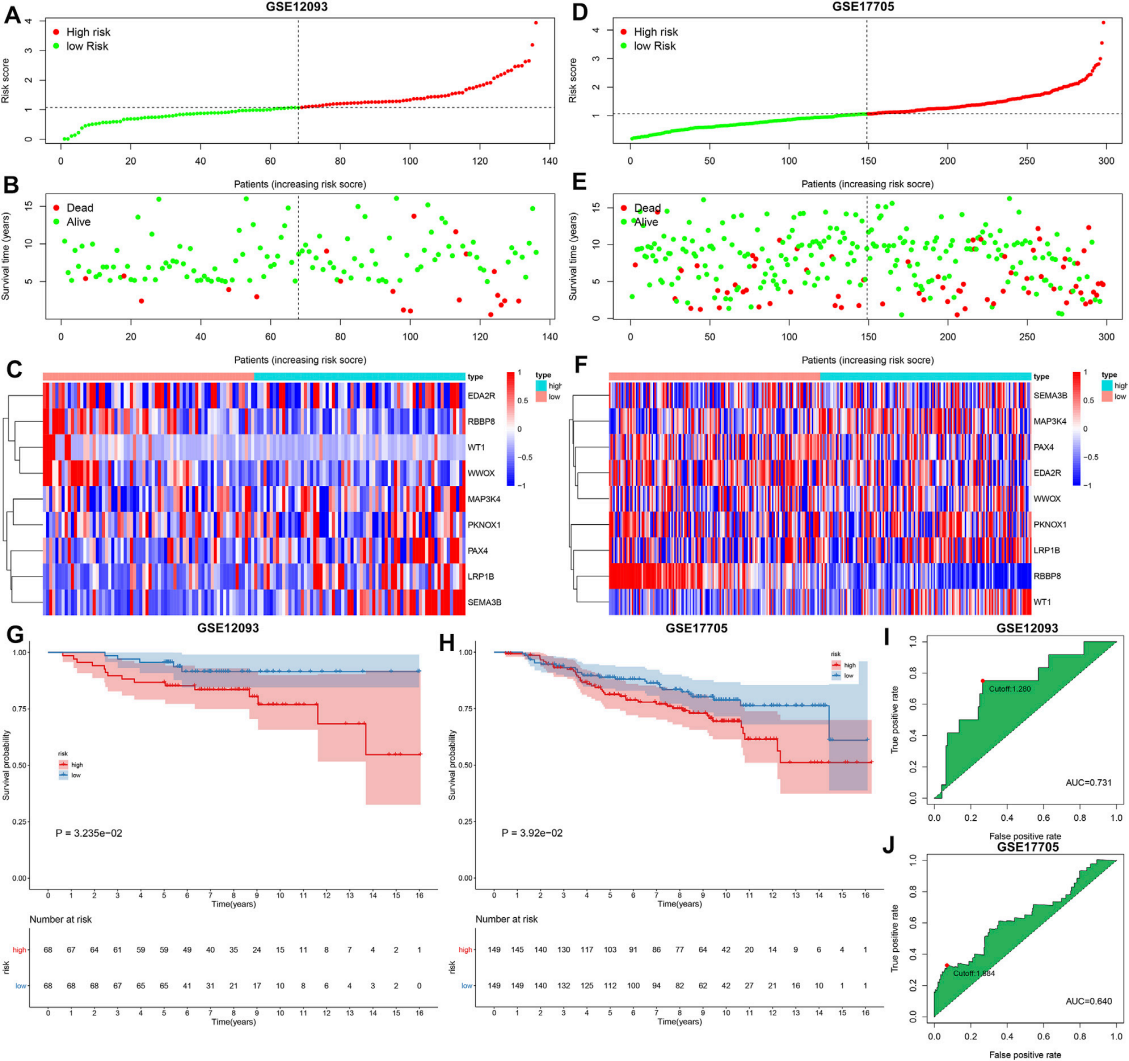
External verification of the predictive performance of the TSG-based prognostic classifier in breast cancer prognosis in the GSE12093 and GSE17705 datasets. **(A–C)** Distribution of RS, survival status, and mRNA expression of characteristic TSGs in high- and low-risk subgroups in the GSE12093 dataset. **(D–F)** Distribution of RS, survival status, and mRNA expression of characteristic TSGs in high- and low-risk subgroups in the GSE17705 dataset. Vertical dotted line represented the cutoff value of high- and low-risk subgroups. **(G,H)** Kaplan–Meier curves of OS and log-rank tests of high- and low-risk subgroups in the GSE12093 and GSE17705 datasets. **(I,J)** ROC curves of the TSG-based prognostic classifier in the GSE12093 and GSE17705 datasets.

The GSE12093 and GSE17705 datasets were curated for externally verifying the performance of the TSG-based prognostic classifier in the prediction of breast cancer prognosis. With the same formula, we determined the RSs of breast cancer patients and stratified patients into high- and low-risk subgroups both in the GSE12093 (Figures 3A–C) and GSE17705 (Figures 3D–F) datasets. Consistent with the results in the discovery set, patients in the high-risk subgroup exhibited poorer OS compared with those in the low-risk subgroup both in the GSE12093 (C-index = 0.670; Figure 3G) and GSE17705 (C-index = 0.607; Figure 3H) datasets. ROC curves uncovered the well predictive efficacy of the TSG-based prognostic classifier in the prediction of breast cancer prognosis both in the GSE12093 (AUC = 0.731 and cutoff = 1.280; Figure 3I) and GSE17705 (AUC = 0.640 and cutoff = 1.884; Figure 3J) datasets.

### Expression and Survival Analysis of Each Ccharacteristic Tumor Suppressor Gene in the Prognostic Classifier

**FIGURE 4:**
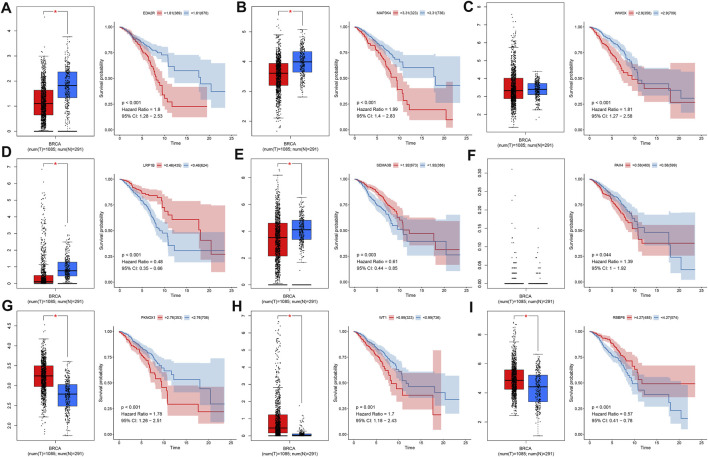
Expression and survival analysis of each characteristic TSG in the prognostic classifier across breast cancer in TCGA dataset. **(A–I)** The mRNA expression of **(A)** EDA2R, **(B)** MAP3K4, **(C)** WWOX, **(D)** LRP1B, **(E)** SEMA3B, **(F)** PAX4, **(G)** PKNOX1, **(H)** WT1, and **(I)** RBBP8 in 1,085 breast cancer tissues and 291 normal breast tissues as well as Kaplan–Meier curves and log-rank tests of their high and low expression groups. *p-value <0.05.

We evaluated the mRNA expression and prognostic significance of each characteristic TSG in the prognostic classifier in breast cancer from TCGA dataset. We observed that EDA2R, MAP3K4, and WWOX exhibited reduced mRNA expression in 1,085 breast cancer tissues compared with 291 normal breast tissues (Figures 4A–C). Meanwhile, high expression of EDA2R, MAP3K4, and WWOX was indicative of unfavorable OS than their low expression. Lower mRNA expression of LRP1B and SEMA3B was detected in breast cancer than normal breast tissues, and their downregulation was in relation to poor clinical outcomes (Figures 4D, E). No significant difference in PAX4 was investigated between breast cancer and normal breast tissues, but its upregulation indicated an undesirable OS for breast cancer patients (Figure 4F). PKNOX1, and WT1 displayed higher mRNA expression in breast cancer tissues in comparison with normal breast tissues as well as their upregulation was associated with poor OS (Figures 4G, H). In Figure 4I, RBBP8 expression was upregulated in breast cancer tissues and patients with its upregulation exhibited the prominent survival advantage.

### Signaling Pathways Underlying the Tumor Suppressor Gene-Based Prognostic Classifier

**FIGURE 5:**
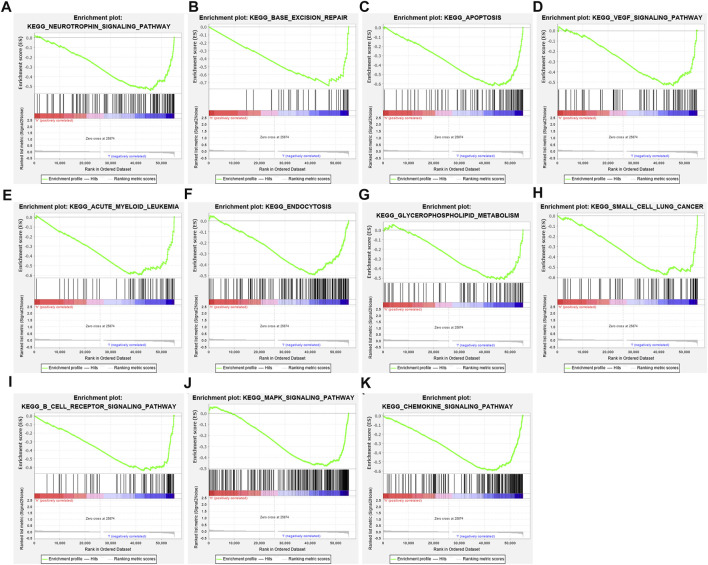
Gene Set Enrichment Analysis (GSEA) identifies signaling pathways underlying the TSG-based prognostic classifier. **(A)** Neurotrophin signaling pathway, **(B)** base excision repair, **(C)** apoptosis, **(D)** VEGF signaling pathway, **(E)** acute myeloid leukemia, **(F)** endocytosis, **(G)** glycerophospholipid metabolism, **(H)** small cell lung cancer, **(I)** B-cell receptor signaling pathway, **(J)** MAPK signaling pathway, and **(K)** chemokine signaling pathway.

GSEA results uncovered that neurotrophin signaling pathway (NES = −2.28, nominal *p*-value <0.0001 and FDR = 0.016), base excision repair (NES = −2.27, nominal *p*-value <0.0001 and FDR = 0.008), apoptosis (NES = −2.22, nominal *p*-value <0.0001 and FDR = 0.007), VEGF signaling pathway (NES = −2.13, nominal *p*-value <0.0001 and FDR = 0.015), acute myeloid leukemia (NES = −2.08, nominal *p*-value <0.0001 and FDR = 0.018), endocytosis (NES = −2.06, nominal *p*-value <0.0001 and FDR = 0.019), glycerophospholipid metabolism (NES = −2.04, nominal *p*-value <0.0001 and FDR = 0.021), small cell lung cancer (NES = −2.03, nominal *p*-value <0.0001 and FDR = 0.019), B cell receptor signaling pathway (NES = −2.01, nominal *p*-value = 0.006 and FDR = 0.021), MAPK signaling pathway (NES = −2.01, nominal *p*-value = 0.006 and FDR = 0.020), and chemokine signaling pathway (NES = −2.00, nominal *p*-value <0.0001 and FDR = 0.019) were significantly activated in low-risk group compared with high-risk group in TCGA dataset (Figures 5A–K).

### Association Between the Tumor Suppressor Gene-Based Prognostic Classifier and Tumor-Infiltrating Immune Cells

**FIGURE 6:**
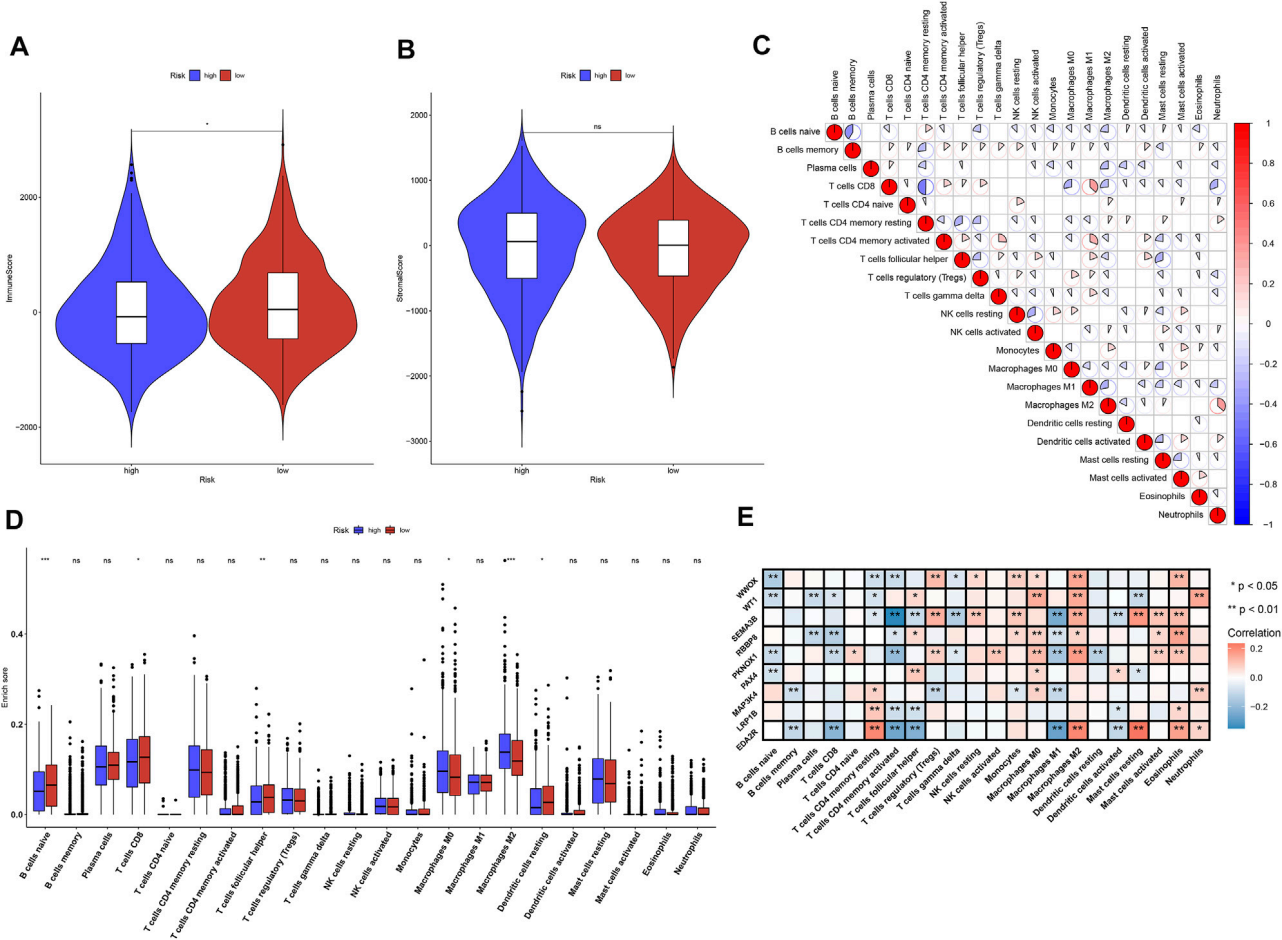
Association between the TSG-based prognostic classifier and tumor-infiltrating immune cells across breast cancer in TCGA dataset. **(A,B)** Comparisons of the immune score and stromal score between high- and low-risk subgroups. **(C)** Spearman’s correlation between the infiltration levels of 22 immune cells in breast cancer specimens. Red, positive correlation; blue, negative correlation. **(D)** Comparisons of the infiltration levels of 22 immune cells between high- and low-risk subgroups. **(E)** Spearman’s correlation between the mRNA expression of characteristic TSGs in the prognostic classifier and the infiltration levels of 22 immune cells across breast cancer specimens. Red, positive correlation; blue, negative correlation. Ns: not significant; *p-value <0.05; **p-value <0.01 and ***p-value <0.001.

Through ESTIMATE computational method, the overall infiltration levels of immune cells and stromal cells were inferred in breast cancer tissues in TCGA dataset. We observed that low-risk samples exhibited increased immune score compared with high-risk samples (Figure 6A), but no significant difference in stromal score was investigated between high- and low-risk specimens (Figure 6B). The infiltration levels of 22 immune cells were estimated in breast cancer tissues *via* CIBERSORT deconvolution algorithm. Spearman’s correlation analysis uncovered the crosstalk between tumor-infiltrating immune cells across breast cancer (Figure 6C). Furthermore, we found that the infiltrating levels of B-cells naïve, T-cells CD8, T-cells follicular helper, and dendritic cells resting were higher in low-risk subgroup compared with high-risk subgroup (Figure 6D). Meanwhile, macrophage M0, and macrophage M2 displayed increased infiltration levels in high-risk subgroup than low-risk subgroup. We also investigated the significant correlation between characteristic TSGs in the prognostic classifier and 22 tumor-infiltrating immune cells across breast cancer (Figure 6E).

### Screening Small Molecule Agents That Potentially Treat Breast Cancer Based on the Tumor Suppressor Genes-Based Prognostic Classifier

**FIGURE 7:**
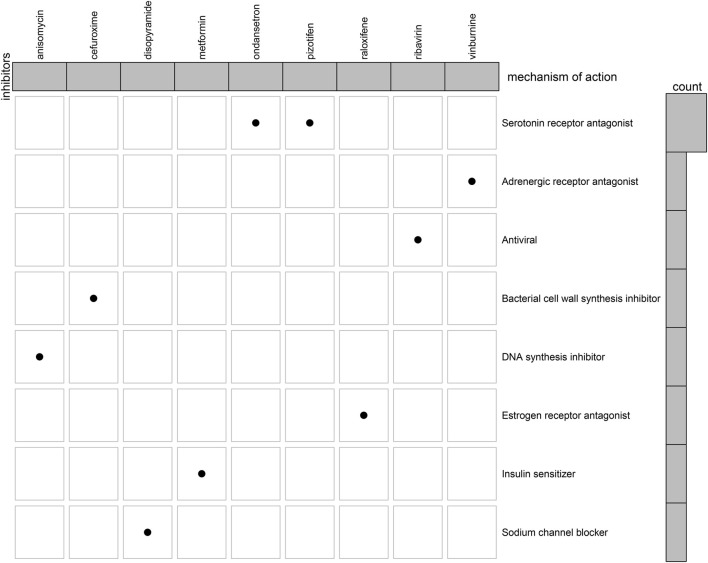
The shared mechanisms among potential small molecule agents by Mode-of-action (MoA).

With adjusted *p*-value <0.05, we screened the top 200 upregulated genes and the top 200 downregulated genes between high- and low-risk subgroups (Supplementary Table S3). Through CMap database, we screened 48 small molecule compounds with a *p*-value <0.05 that might potentially treat breast cancer based on the TSG-based prognostic classifier (Table 3). The shared mechanisms among small molecule compounds were evaluated through MoA. In Figure 7, we observed ondansetron and pizotifen shared serotonin receptor antagonist.

**TABLE 3:** Prediction of potential small molecule agents based on the TSG-based prognostic classifier by connectivity map (CMap).

Cmap name (cell line)	Mean	N	Enrichment	*p*-Value	Specificity	Percent non-null
Raloxifene (MCF7)	0.766	3	0.971	0.00004	0	100
Thapsigargin (MCF7)	0.711	2	0.979	0.00076	0.0915	100
Wortmannin (HL60)	0.504	4	0.842	0.00101	0.0065	75
Oxolinic acid (PC3)	0.696	2	0.972	0.00123	0	100
Securinine (MCF7)	−0.748	2	−0.971	0.00167	0.0133	100
PHA-00767505E (PC3)	−0.695	2	−0.971	0.00169	0	100
Chlorphenamine (MCF7)	0.628	2	0.964	0.00217	0	100
Iobenguane (MCF7)	0.653	2	0.959	0.00286	0	100
Disopyramide (MCF7)	−0.644	2	−0.959	0.00372	0.0319	100
Naltrexone (PC3)	0.635	2	0.95	0.00467	0.0071	100
Josamycin (PC3)	0.591	2	0.949	0.00477	0	100
0198306-0000 (MCF7)	0.631	2	0.946	0.00545	0.0063	100
Rifabutin (MCF7)	0.619	2	0.946	0.00555	0.0266	100
16-Phenyltetranorprostaglandin E2 (MCF7)	0.574	2	0.946	0.00557	0.0071	100
Pirenzepine (PC3)	−0.597	2	−0.945	0.00658	0.021	100
Prestwick-1103 (MCF7)	−0.589	2	−0.94	0.00759	0.0217	100
Pirinixic acid (PC3)	0.542	2	0.934	0.00833	0.0274	100
Atropine oxide (MCF7)	0.534	2	0.931	0.00909	0.0121	100
Helveticoside (PC3)	−0.605	2	−0.933	0.00919	0.1039	100
Ondansetron (MCF7)	−0.597	2	−0.932	0.0095	0.0286	100
Vinburnine (MCF7)	−0.611	2	−0.931	0.00996	0.0301	100
Withaferin A (MCF7)	0.476	2	0.925	0.01099	0.1217	100
Paclitaxel (PC3)	0.5	2	0.924	0.01117	0	100
Ornidazole (MCF7)	−0.514	2	−0.926	0.01131	0.0168	100
Benzamil (MCF7)	−0.301	3	−0.814	0.01284	0.0072	66
Tinidazole (PC3)	0.461	2	0.915	0.01429	0.016	100
Nicardipine (MCF7	0.463	2	0.915	0.01433	0.0127	100
Decamethonium bromide (MCF7)	−0.22	2	−0.915	0.01459	0	50
Naftidrofuryl (MCF7)	−0.334	2	−0.897	0.02086	0.0333	50
Iloprost (MCF7)	−0.295	2	−0.897	0.02107	0.0248	50
0317956-0000 (PC3)	0.414	4	0.683	0.02166	0.0078	75
Sulfamonomethoxine (MCF7)	−0.31	2	−0.889	0.02443	0.057	50
Halofantrine (MCF7)	−0.202	2	−0.888	0.02491	0.021	50
Carcinine (MCF7)	0.308	2	0.889	0.02565	0	50
Celecoxib (MCF7)	0.374	4	0.665	0.02851	0.0106	75
Moroxydine (MCF7)	−0.323	2	−0.875	0.03135	0.0839	50
Ergocalciferol (MCF7)	−0.379	2	−0.874	0.03155	0.0073	50
5248896 (MCF7)	0.364	2	0.864	0.03744	0.05	50
15-Delta prostaglandin J2 (MCF7)	0.27	8	0.469	0.03749	0.521	50
Cyproheptadine (MCF7)	0.355	2	0.864	0.0376	0.0255	50
Stachydrine (MCF7)	−0.309	2	−0.856	0.04151	0.0733	50
Spiradoline (MCF7)	−0.302	2	−0.854	0.04241	0.0694	50
Amantadine (MCF7)	−0.301	2	−0.854	0.04255	0.102	50
Trimethadione (MCF7)	−0.244	2	−0.853	0.04288	0.025	50
Imipenem (MCF7)	0.286	2	0.853	0.0434	0.0143	50
Metformin (MCF7)	0.314	7	0.489	0.04537	0.0827	57
Salsolinol (MCF7)	0.281	2	0.846	0.04783	0.0357	50
Bupivacaine (MCF7)	−0.362	2	−0.843	0.04927	0.0136	50

### Establishment of Five Tumor Suppressor Gene-Based Subtypes With Different Clinical Outcomes

**FIGURE 8:**
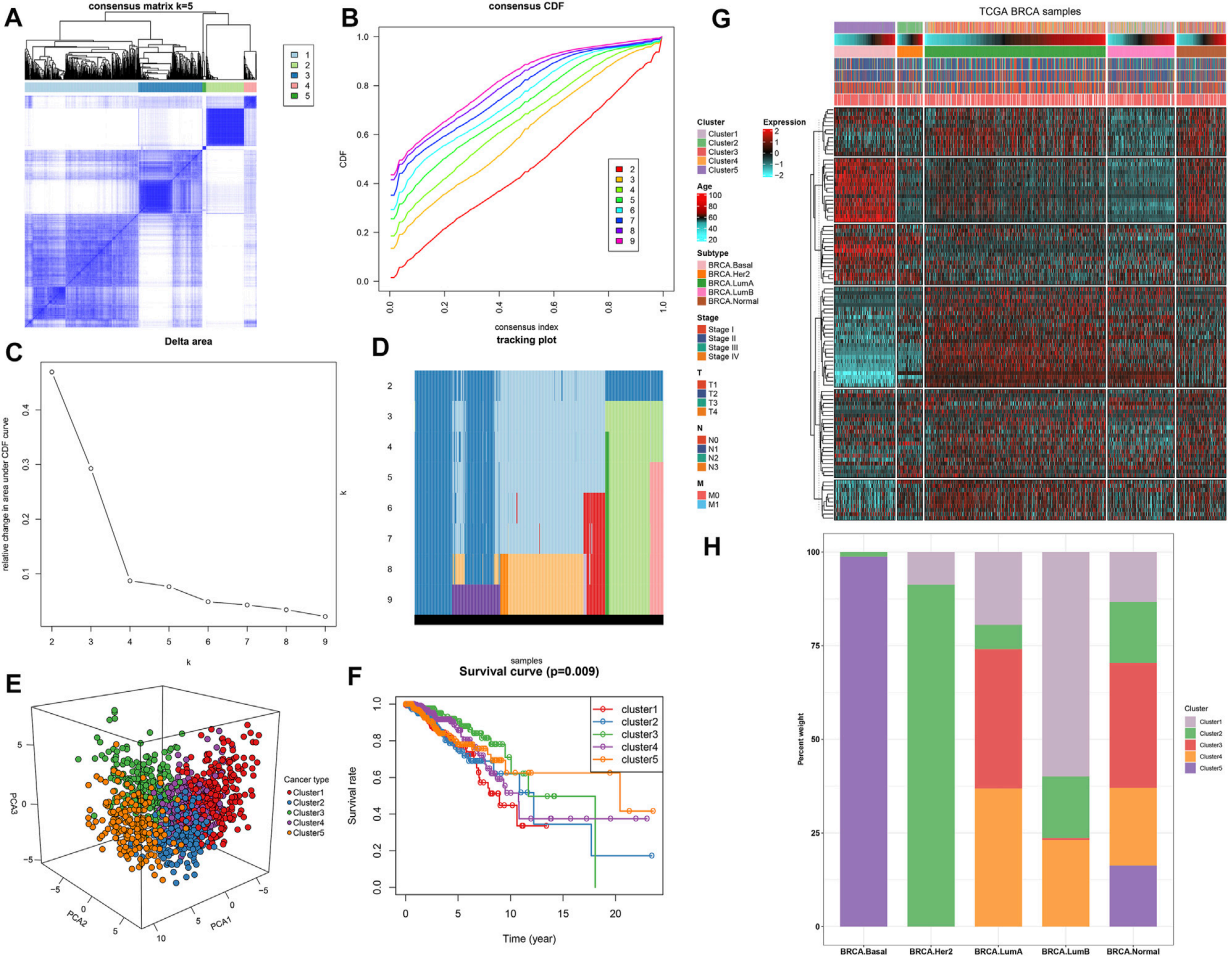
Establishment of five TSG-based subtypes with different clinical outcomes across breast cancer in TCGA dataset. **(A)** Consensus matrix when *k* = 5. **(B)** Consensus CDF diagram for the consensus distribution when *k* = 2–9. **(C)** Delta area under CDF curves when *k* = 2–9. **(D)** Item tracking plot for the consensus clustering of items corresponding to each k. **(E)** Principal component analysis (PCA) plots for the difference among five clusters based on the mRNA expression of the top 100 TSGs with the most variance. **(F)** Kaplan–Meier curves and log-rank test of five clusters. **(G)** Heat map visualizing the clinical phenotypes in five clusters. **(H)** Comparison of five TSG-based subtypes and known breast cancer subtypes.

Based on the mRNA expression profiling of the top 100TSGs with the most variance, breast cancer patients in TCGA dataset were clustered into five clusters, named as clusters 1–5 (Figures 8A–D). PCA results confirmed the prominent difference among the five clusters (Figure 8E). Survival analysis uncovered the significant survival difference among the five clusters (Figure 8F). Among them, cluster 1 displayed the poorest clinical outcomes. Figure 8G depicts the heterogeneity in clinical phenotypes including age, T, N, M, stage, and known breast cancer subtypes among the five clusters. Considering the known breast cancer subtypes, we compared the five TSG-based subtypes with known breast cancer subtypes (Basal, Her2, LumA, and LumB). Our results showed that specific TSG-based subtypes had a high coincidence rate with known subtypes (Figure 8H). Especially, TSG-based subtype 5 was highly coincident with Basal subtype, and TSG-based subtype 2 displayed a high coincidence with Her2 subtype, indicating that TSG-based molecular subtypes had certain accuracy and stability, and patients with TSG-based subtypes 2 and 5 could separately receive similar treatment as patients of Basal subtype and Her2 subtype.

## Discussion

Development of early detection, diagnostic and therapeutic strategies has led to a continuous decline in the mortality of breast cancer patients, metastatic patients usually display an undesirable prognosis even though with multimodal treatments (Liang et al., 2020). The thought-provoking research has highlighted the importance of applying innovative methods for identifying high-risk patients and improving the management of the patient (Zhang D et al., 2020). With the development of personalized medicine, gene expression profiling plays important roles in offering guidance for personalized therapy optimization. In our research, we developed a TSG-based prognostic classifier for breast cancer. Following verification, this prognostic classifier may robustly predict the clinical outcomes of the patients.

Herein, we observed abnormal expression and dysfunction of each characteristic TSG in the TSG-based prognostic classifier. EDA2R, a tumor necrosis factor receptor, is downregulated in breast cancer through promoter methylation, which binds to ectodysplasin-A2 and induces cell deaths (Punj et al., 2010). As a tumor suppressor, EDA2R prevents malignant transformation and cancer progression (Tanikawa et al., 2009). LRP1B mutation contribute to favorable response to immunotherapy across pan-cancer (Brown et al., 2021). MAP3K4 maintains epithelial-mesenchymal transition in trophoblast stem cells, which potentially contributes to breast cancer (Abell et al., 2011). MAP3K4 can be predictive of preoperative radiotherapeutic responses for locally advanced breast cancer (Tanic et al., 2018). PAX4, a transcriptional modulator, modulates metastasis of epithelial cancers (Zhang et al., 2015). PKNOX1 is involved in modulating breast adenocarcinoma progression (Fernandez et al., 2008). RBBP8 predisposes to early-onset breast cancer progression (Zarrizi et al., 2020). SEMA3B, a secreted axonal guidance molecule, suppresses breast cancer development and metastasis (Shahi et al., 2017). WT1 plays an oncogenic role in breast cancer pathogenesis (Zhang Y et al., 2020). Evidence suggests the inhibitory role of WWOX tumor suppressor gene in breast cancer (Pospiech et al., 2018). We further investigated signaling pathways underlying the TSG-based prognostic classifier. Carcinogenic pathways and immune-related pathways including neurotrophin signaling pathway, base excision repair, apoptosis, VEGF signaling pathway, acute myeloid leukemia, endocytosis, glycerophospholipid metabolism, small cell lung cancer, B-cell receptor signaling pathway, MAPK signaling pathway, and chemokine signaling pathway were prominently activated in the low-risk group, indicative of the critical biological implication of the TSG-based prognostic classifier.

Immunotherapeutic strategies have been included in the standard of care for a variety of human cancers. The evidence emphasizes the importance of tumor-infiltrating immune cells in the host anti-cancer immune responses in the natural course of breast cancer (Zhang and Plitas, 2021). Our results that B cells naïve, T cells CD8, T cells follicular helper, and dendritic cells resting displayed higher infiltration levels in low-risk subgroup than high-risk subgroup. Meanwhile, macrophage M0, and macrophage M2 had increased infiltration levels in high-risk subgroup than low-risk subgroup. This indicated that the prognostic classifier can be utilized for prediction of tumor immune infiltration. Moreover, we screened 48 small molecule agents that may potentially treat breast cancer based on the TSG-based prognostic classifier. Especially, ondansetron and pizotifen shared serotonin receptor antagonist. For instance, ondansetron may alleviate chemotherapy-induced nausea and vomiting in breast cancer (Yeo et al., 2020). Furthermore, evidence suggests that pizotifen suppresses proliferative and migratory capacities of gastric cancer as well as colon cancer (Piao and Shang, 2019; Jiang et al., 2020). The anti-breast cancer effect of pizotifen will be verified in more experiments.

Except for developing a clinical indicator regarding TSGs, we constructed clinically relevant classification of breast cancer based on the top 100 TSGs with the most variance. In general, patients who have similar clinicopathological features exhibit much heterogeneity in prognosis. Here, five TSG-based subtypes were clustered, with different prognosis and clinicopathological features. Thus, comprehensive indicators from single TSGs may prominently ameliorate survival outcomes. Nevertheless, there are several limitations in our study. First, this was a retrospective study according to appropriate mRNA expression profiles and prognostic data of breast cancer patients. The predictive performance of the TSG-based prognostic classifier should be verified in a prospective cohort. Second, the mechanisms underlying prognosis-relevant TSGs regulate breast cancer pathogenesis requires further experimental verification for improvement of the present therapeutic practice of breast cancer.

## Conclusion

Collectively, a novel prognostic classifier on the basis of TSG expression profiling was established for breast cancer and externally verified in the two cohorts. The prognostic classifier possessed the potential to predict breast cancer prognosis as well as tumor immune infiltration. Moreover, we screened promising small molecule agents against breast cancer. The predictive performance of the prognostic classifier will be verified in prospective cohorts in our future research.

## Data Availability

The original contributions presented in the study are included in the article/Supplementary Material, further inquiries can be directed to the corresponding author.

## Supplementary Material

Supplementary Table 1 The list of 1217 tumor suppressor genes.

Supplementary Table 2 The 116 prognosis-related TSGs for breast cancer patients in TCGA cohort.

Supplementary Table 3 The top 200 up-regulated genes and the top 200 downregulated genes between high- and low-risk subgroups.

## Abbreviations

AUC, area under the curve; CI, confidence interval; CIBERSORT, Cell type Identification By Estimating Relative Subsets Of RNA Transcripts; CMap, Connectivity map; ESTIMATE, Estimation of STromal and Immune cells in MAlignant Tumors using Expression data; FDR, false discovery rate; GEO, Gene Expression Omnibus; GSEA, Gene Set Enrichment Analysis; KEGG, Kyoto Encyclopedia of Genes and Genomes; MoA, Mode-of-action; NES, normalized enrichment score; OS, overall survival; PCA, principal component analysis; RS, risk score; ROC, receiver operating characteristic; TSGs, tumor suppressor genes; TSGene, Tumor Suppressor Gene database; TCGA, The Cancer Genome Atlas.
